# *Brevibacillus laterosporus*: A Probiotic with Important Applications in Crop and Animal Production

**DOI:** 10.3390/microorganisms12030564

**Published:** 2024-03-12

**Authors:** Yucheng Liu, Xueying Zai, Guangying Weng, Xianyong Ma, Dun Deng

**Affiliations:** 1 Institute of Animal Science, Guangdong Academy of Agricultural Sciences, State Key Laboratory of Swine and Poultry Breeding Industry, Key Laboratory of Animal Nutrition and Feed Science in South China, Ministry of Agriculture and Rural Affairs, Guangdong Provincial Key Laboratory of Animal Breeding and Nutrition, Guangdong Engineering Technology Research Center of Animal Meat Quality and Safety Control and Evaluation, Guangzhou 510640, China; liuyucheng4934@outlook.com (Y.L.); zxying214@163.com (X.Z.); wengguangying123@163.com (G.W.); 2 College of Animal Science & Technology, Zhongkai University of Agriculture and Engineering, Guangzhou 510225, China

**Keywords:** *Brevibacillus laterosporus*, probiotic mechanisms, crop cultivation, animal production, antimicrobial peptides

## Abstract

*Brevibacillus laterosporus* (*B. laterosporus*) is widely distributed in nature and demonstrates significant potential for applications in biological control, environmental protection, agricultural production, and clinical medicine. This review provides a comprehensive overview of the applications of *B. laterosporus* in crop cultivation and animal feeding, as well as an examination of the antimicrobial peptides produced by *B. laterosporus* and their antibacterial mechanisms. *B. laterosporus* enhances crop cultivation by secreting hydrolases to improve nutrient absorption capabilities, synthesizing hormones to promote crop growth, and producing proteins to inhibit the reproduction of harmful organisms. *B. laterosporus* has been used to improve animal production by regulating the structure of the intestinal microbiota and inhibiting the growth of pathogenic bacteria through the secretion of various antimicrobial peptides. The bactericidal activity of Brevilaterins secreted by *B. laterosporus* is attributed to their ability to bind to lipopolysaccharide/lipid II molecules on the cell membrane, thereby altering permeability. Brevilaterins also inhibit bacterial reproduction by affecting relevant gene pathways in the cell membranes of pathogenic bacteria. These pathways include ATP synthesis, peptidoglycan biosynthesis, membrane transport, and cellular metabolism. In conclusion, *B. laterosporus* exhibits substantial potential as a probiotic activity in crop and animal production. However, applications of *B. laterosporus* in animal production could be improved, necessitating further research to elucidate the underlying probiotic mechanisms.

## 1. Introduction

*Brevibacillus laterosporus* (*B. laterosporus*) is widely distributed and can be found in a variety of ecosystems, including soil, water, and animal bodies [1,2,3,4]. Precisely because of its wide range of sources, researchers have obtained strains with different characteristics from different places such as the rhizosphere of plants, seawater, and animal digestive systems. *B. laterosporus* demonstrates adaptability to varying conditions of temperature and pH [5]. The growth of *B. laterosporus* commences at 4 °C, and it exhibits a high reproductive capacity within the temperature range of 15 °C to 37 °C. Moreover, *B. laterosporus* demonstrates excellent pH tolerance and adaptation in the range of 3.0 to 7.0. *B. laterosporus* (Firmicutes, *Bacilli*, *Bacillales*, *Paenibacillaceae*, *Brevibacillus*) is a facultative anaerobic bacterium that produces rod-shaped cells and endospores. *B. laterosporus* is significantly different from other *Bacillus* species due to its unique canoe-shaped parasporal bodies [6]. Some *B. laterosporus* strains produce cytoplasmic crystalline inclusions of various shapes and sizes.

*B. laterosporus* was initially identified in water by Laubach in 1916 and named *Bacillus laterosporus* [7]. The morphological and physiological characteristics of *B. laterosporus* were meticulously elucidated by Laubach. *B. laterosporus* exhibits a granular morphology with rounded ends and possesses globular spores. Its dimensions range from (0.375–0.5) μm × (1.125–4) μm, which is shorter than the majority of *Bacilli*. Due to limitations in the identification technology available at that time, its characteristics were described as similar to those of *Bacillus*, leading to its subsequent designation as *Bacillus laterosporus*. In 1996, Shida employed the analysis of 16S rRNA gene sequences and phylogenetics to demonstrate that *Bacillus brevis* (*B. brevis*) clusters exhibited distinct phylogenetic separation from other *Bacillus* clusters [8]. Therefore, Shida proposed reclassifying the *B. brevis* cluster as a novel genus named *Brevibacillus* gen. nov., which includes *Bacillus laterosporus*, resulting in the official renaming of *Bacillus laterosporus* as *Brevibacillus laterosporus*.

*B. laterosporus* is a probiotic bacterium exhibiting diverse biological activities encompassing insecticidal, antibacterial, anti-tumor, and biodegradation properties [9]. It has applications in crop cultivation, animal production, clinical medicine, and biological degradation. *B. laterosporus* is widely used to promote growth and protect crops. By producing growth hormones, *B. laterosporus* stimulates root development and improves nutrient-uptake efficiency, thereby increasing crop yield and quality. *B. laterosporus* also demonstrates inhibitory activity against a variety of crop pests and pathogens, thereby effectively regulating harmful organisms. *B. laterosporus* plays an important probiotic role in animal feeding. By regulating the balance of the intestinal tract, *B. laterosporus* improves digestive function and promotes animal growth. The scope of applications for *B. laterosporus* also encompasses the medical field. The antimicrobial peptides (AMPs) [10,11] and enzymes [12,13] produced by *B. laterosporus* possess significant medicinal value. Moreover, *B. laterosporus* also exhibits the capability to synthesize anticancer substances such as Brevilaterin B [14]. The degradation of various substances by *B. laterosporus* has been increasingly observed, exemplified prominently by the conversion of polyvinyl alcohol into acetate. The utilization of *B. laterosporus* in various domains demonstrates its significant value and extensive prospects. Microorganisms play a pivotal role in promoting sustainable agricultural development, finding extensive applications in microbial pesticides, microbial fertilizers, and microbial feeds. *B. laterosporus* not only exhibits remarkable insecticidal and antibacterial properties but also contributes significantly to soil enhancement. The multifunctionality demonstrated by *B. laterosporus* has captured our attention, particularly the potential application of its AMPs as alternatives to antibiotics in animal breeding. Consequently, we have chosen to concentrate our research on *B. laterosporus* within the domains of crop cultivation and animal production. The focus of this study will focus on the probiotic role of *B. laterosporus* in animal production and the involvement of probiotic activity of *B. laterosporus* in crop symbiosis, as well as exploring the mechanism of action of antimicrobial peptides produced by *B. laterosporus*.

## 2. The Salutary Effects of *B. laterosporus* on Crops

### 2.1. Enhances Nutrient Acquisition and Promotes Crop Growth

Microorganisms form a symbiotic relationship with plants, providing essential elements and synthetic hormones through metabolites, thereby significantly enhancing crop yield [15,16]. The metabolites produced by microorganisms play a crucial role in providing plants with essential elements, such as organic nitrogen and phosphorus, thereby addressing the issue of inaccessible inorganic nutrients in the soil. The application of microbial hormones facilitates an increase in leaf area, prolongs the rate of senescence, induces flower bud differentiation, and enhances fruit expansion.

The effectiveness of *B. laterosporus* in enhancing nutrient acquisition is significant. It promotes the absorption and utilization of mineral nutrients by crops by secreting phosphatase to convert organic phosphorus into inorganic phosphorus in the soil [17,18]. *B. laterosporus* (rhizosphere of Lithocarpus sundaicus) isolated from the Sangagana forest dissolved 27.67 mg/L of inorganic phosphorus [19]. This phosphate-solubilizing ability is closely related to the phosphate-solubilizing genes in its genome. The strain *B. laterosporus* K75, which exhibits robust capabilities in solubilizing phosphate, harbors additional genes (pqq, pstA, and pstB) that are associated with the process of phosphate solubilization [20].

Furthermore, *B. laterosporus* has the capability to biosynthesize hormones. For example, *B. laterosporus* SVC(II)14 isolated from the rhizosphere soil of a Haryana cotton-growing area synthesized 4.74 μg/mL of indole acetic acid (IAA) [21]; Świątczak reported that *B. laterosporus* K75 exhibited the highest concentration of synthesized IAA, totaling 13.892 µg/mL [20]. Furthermore, particular strains have demonstrated the ability to produce up to 45.77 µg/mL of IAA under optimized culture conditions, such as with tryptophan supplementation [22]. The different IAA-synthetic abilities of *B. laterosporus* have been attributed to variations in its genome. For instance, the high plant hormone-producing strain K75 harbors additional IAA synthesis genes (trp A, B, C, D, E, F, and S) within its genome [20].

The practical applications of *B. laterosporus* encompass the effective stimulation of crop root growth and enhancement of their absorptive capacity, thereby resulting in augmented crop yields [20,22]. Some studies have shown that *B. laterosporus* significantly increased the biomass of crops [20,23], as reflected by increased leaf weight and root length in maize by 54.55% and 26.90%, respectively, and an increase in the fresh weight of potato by 6.80%.

### 2.2. Suppresses Reproduction of Harmful Organisms

#### 2.2.1. Inhibits Pathogenic Fungi in Crops

The cultivation of crops often encounters challenges posed by fungal diseases, resulting in significant financial losses for agricultural practitioners. Thus, the use of microorganisms as a viable solution to inhibit the growth and reproduction of fungi has emerged [24]. These beneficial microorganisms enter the soil or plant and compete with potentially pathogenic fungi for nutrients, space, and resources, thereby limiting fungal reproduction and infection. This pollution-free, sustainable, and efficient biocontrol method has gained extensive applications in agricultural production and crop protection. *B. laterosporus* strains can control or inhibit the reproduction of harmful fungi, such as *Phytophthora capsici* [25], *Botryosphaeria dothidea* [26], and *Fusarium oxysporum* [20], thereby promoting crop growth [27,28] (Table 1).

#### 2.2.2. Inhibition of Pathogenic Bacteria in Crops

*B. laterosporus* inhibits a variety of bacteria that infect crops [31]. For example, the control effect of tomato bacterial wilt caused by *Ralstonia solanacearum* was 58.42–68.68% when *B. laterosporus* X10 was added to tomatoes [32]. The biocontrol efficiency of *B. laterosporus* AMCC100017 against potato common scab (PCS) caused by Streptomyces bottropensis was 70.5% [23]. The biocontrol efficacy of *B. laterosporus* BL12 against PCS was determined to be 34.29% [33]. Some beneficial bacteria (*Pseudomonas* and *Microbacterium*) related to the control of crop diseases are significantly positively correlated with *B. laterosporus* BL12 and significantly negatively correlated with the disease index, which may cooperate with BL12 to control PCS [33]. The biocontrol efficiency of *B. laterosporus* SN19-1 against bacterial leaf blight caused by *Xanthomonas* was 90.92% [34]. The inhibitory effect of *B. laterosporus* B4 on *Acidovorax avenae* subsp. *avenae* was significant, with a biocontrol efficacy of 71.9% against brown streak disease caused by *Acidovorax avenae* subsp. *Avenae* [35]. The mechanism of action may be that *B. laterosporus* B4 disrupts the formation of the pathogenic bacterial biofilm by producing bacteriocins that directly inhibit growth or change the expression of virulence-related genes, leading to the leakage of intracellular substances.

#### 2.2.3. Inhibits Pests in Crops

The pathogenicity of *B. laterosporus* has been extensively documented against parasitic nematodes, mollusks, as well as eggs and larvae of various insect orders including Coleoptera, Diptera, and Lepidoptera [36]. Nematodes are common crop pathogens that cause severe damage. Beneficial microbial agents inhibit nematodes by promoting defense responses through parasitization, occupation, and killing [37]. *B. laterosporus* kills nematodes by inhibiting egg hatching and larval development [38]. The culture supernatant of *B. laterosporus* F5 demonstrated significant efficacy against microworms and *Meloidogyne incognita*, leading to a larval mortality rate of 90% [39]. Notably, the median lethal concentration (LC_50_) for *Meloidogyne incognita* was 0.4 mg/mL. The culture supernatant of *B. laterosporus* G4 contained an alkaline protease (designated BLG4) that exhibited potent nematicidal activity. Scanning electron microscopy has revealed that BLG4 caused severe damage to the nematode cuticle, which subsequently underwent digestion by the host, suggesting that hydrolytic proteases may serve as a key toxic component in nematode eradication [40,41].

*B. laterosporus* also has efficacy against a variety of insects, including Lepidoptera, Diptera, and Coleoptera [36]. *B. laterosporus* V12/001946 isolated from hybrid cabbage seed had a significant killing effect on a variety of Lepidopteran pests at a concentration of 10^10^ cells/mL. The brood mortality rates of *Epiphyas postvittana*, *Cnephasia jactatana*, and *Cydia pomonella* were 73.3%, 76.7%, and 60%, respectively [36]. The dipteran pests can be eradicated by the lethal effects of *B. laterosporus*. For example, Rivers et al. showed that *B. laterosporus* LMG15441 has a 100% mortality rate against *Aedes albopictus* larvae [42]. The lethal concentrations of *B. laterosporus* UNISS18 to *Culex pipiens*, *Aedes aegypti*, *Calliphora vomitoria*, and *spotted wing Drosophila* were 0.10, 0.18, 78.84, and 217.51 × 10^6^ spores/mL, respectively [43]. *B. laterosporus* Bon707 demonstrated a lethality rate of 70.5% against *Chrysomya megacephala* at a concentration of 1.46 × 10^7^ CFU/mL [44].

The toxicity of particular *B. laterosporus* strains towards insects is attributed to various factors, including spores, cell crystals, and the toxic proteins they produce [45]. The fermentation supernatants of *B. laterosporus* MB438 and MB439 exhibited insecticidal activity against *Diabrotica virgifera* in maize roots and leaves, and two toxin proteins were subsequently identified from these organs [46]. Following isolation, a strain of *B. laterosporus* EG5553, obtained from grain, was found to harbor Mpp75Aa1, an insecticidal protein. This protein can undergo oligomerization through corn rootworm protease processing and subsequently bind to receptors on the midgut membrane, resulting in perforation and tissue damage that ultimately leads to the mortality of corn rootworm [47].

## 3. The Probiotic Effects of *B. laterosporus* on Animals

### 3.1. Regulation of Animal Growth

Utilizing microbial agents as a means of regulating animal growth offers numerous benefits. Microbial agents effectively enhance the functionality of the digestive system, thereby optimizing nutrient absorption and feed utilization. Additionally, microbial agents facilitate the accelerated growth and reproduction of farmed animals, resulting in shortened breeding cycles and improved efficiency. Moreover, microbial agents stimulate and regulate the immune system to bolster resistance against external environmental factors and pathogenic microorganisms, thereby mitigating the risk of infection and death. *B. laterosporus* has been added to animal feed, and the improvements in the growth of farmed animals are shown in Table 2.

Supplementation of the broiler diet with *B. laterosporus* S62-9 resulted in a significant 7.2% increase in broiler weight, accompanied by a remarkable reduction of 5.19% in the feed conversion rate and enhancement of the immune response [48]. Adding *B. laterosporus Texasporus* to the diet of broilers infected with *Salmonella* effectively counteracted the decline in performance and the increase in the immune factor IgM induced by the pathogenic bacteria [49]. Additionally, adding *B. laterosporus* S62-9 to feed enhanced the pH level, brightness, and tenderness of the chicken meat, as well as increasing its protein and fat contents, thereby improving the quality and flavor of the broiler chickens [52].

*B. laterosporus* has also been used in the aquaculture of fish, crabs, shrimp, and freshwater turtles and has demonstrated significant benefits, such as the promotion of growth, antibacterial properties, and enhancement of body weight [53]. Introducing *B. laterosporus* PBC01 into the water significantly enhanced the growth performance of crucian carp [50]. The inclusion of *B. laterosporus* in aquaculture water significantly enhanced the antioxidant status of crucian carp in both serum and liver, while also stimulating the activities of intestinal digestive enzymes in crucian carp. The growth and health status of crucian carp can be significantly enhanced by supplementation with an appropriate dosage of *B. laterosporus* as a water probiotic [50]. Feeding *Litopenaeus vannamei* a diet containing 10^7^ CFU of *B. laterosporus* FAS05 per gram significantly increased its specific growth rate and reduced the immune activity of hemocyte reactive oxygen species in a 28-day culture experiment [51]. Taken together, these results indicate that using *B. laterosporus* as a feed additive in various cultured species significantly enhances growth and bolsters the immune response.

### 3.2. Regulation of Intestinal Health

The intestinal microbiota plays a crucial role in the maintenance of overall health and homeostasis [54]. The gut microbiota actively participates in food decomposition and nutrient absorption by synthesizing enzymes that facilitate the breakdown of carbohydrates, proteins, and fats into bioavailable forms for utilization. These processes enhance digestive efficiency and facilitate the augmentation of energy reserves, thereby bolstering productivity. In addition, the gut microbiota modulates the immune response by stimulating the activation of T cells, B cells, natural killer cells, and other immune cells, thereby enhancing their ability to recognize and eliminate pathogens, and bolstering the defense mechanism.

The regulation of the intestinal flora structure by *B. laterosporus* enhances disease resistance, promotes the relative abundance of beneficial bacteria, and inhibits the proliferation of harmful microorganisms. Feeding with *B. laterosporus* S62-9 significantly enhanced the relative abundance of beneficial bacteria (*Adlercreutzia*, *Akkermansia*, *Lactobacillus*, *Intestinimonas*, and *Ruminococcus*) in the broiler cecum while reducing the relative abundance of harmful bacteria (*Pseudomonas*, *Klebsiella*, *Cupriavidus*, and *Ralstonia*) [48]. *B. laterosporus* sustains intestinal immune function by competing with pathogenic microorganisms on the mucosa to inhibit colonization and promote intestinal health. Feeding *B. laterosporus* BL1 to a high-fat mouse model improved the structure of the intestinal flora by increasing the number of beneficial bacteria (*norank_f_Muribaculaceae*) and reducing the abundance of pro-inflammatory bacteria (*Faecalibaculum*) [55]. In the aflatoxin B1 (AFB_1_)-infected quail model, the number of *Escherichia coli* increased while the number of lactic acid bacteria decreased in the ileum of quail fed AFB_1_ [56]. However, this effect was reversed by adding *B. laterosporus* Bl (*p* < 0.001). Therefore, these results suggest that the probiotic *B. laterosporus* improves the gut’s microbial-community structure in different animals, thereby promoting overall health.

### 3.3. Other Functions

An existing patent has identified that a naturally prepared *B. laterosporus* Texasporus (BT) lipopeptide derived from *B. laterosporus Texasporus* exhibits significant efficacy in the treatment of obesity and associated disorders [53]. Examples of BT lipopeptide inventions include oral administration of one or more BT lipopeptides, which effectively reduced body weight in obese patients, controlled blood glucose level, and enhanced insulin sensitivity; treatment with BT peptides decreased visceral adiposity and improved fatty liver in a mouse obesity model after 30 days of feeding [53]. Weng reported that *B. laterosporus* BL1 effectively reduced weight and fat in obese mice fed a high-fat diet (HFD), while also lowering blood lipid and glucose levels, thereby achieving less adipose tissue [55]. A HFD group exhibited a significant increase in body weight and body fat content compared to the CON group (*p* < 0.001), whereas *B. laterosporus* BL1-fed obese mice demonstrated remarkable reductions of 41.26% and 33.39% in body weight and body fat content, respectively (*p* < 0.01) [55]. These findings suggest that *B. laterosporus* possesses a probiotic function that effectively mitigates obesity and lipid accumulation in animals.

## 4. *B. laterosporus* Antimicrobial Peptides

### 4.1. Structural Features of Brevilaterins

The innate immune system encompasses a crucial class of small peptides known as AMPs, which are widely distributed in nature. AMPs exhibit a broad spectrum of inhibitory efficacy against bacteria, fungi, and viruses [57,58] and play crucial roles as innate immune effectors across diverse species, including humans, animals, and crops. AMPs serve as the first line of defense against foreign invaders [59,60]. AMPs possess diverse biological functions [61]. AMPs exhibit broad-spectrum antimicrobial properties by targeting a wide range of pathogens, such as bacteria, fungi, viruses, and parasites. They disrupt microbial cell membranes, inhibit intracellular processes, and modulate immune responses to combat infections. AMPs regulate the immune system by affecting the activation, migration, and function of immune cells, such as monocytes, macrophages, neutrophils, and T cells. Particular AMPs promote wound healing by enhancing angiogenesis during tissue regeneration and re-epithelialization. The immunomodulatory effects of AMPs help to mitigate infection-related complications during healing. AMPs exhibit anticancer activity by inducing apoptosis (programmed cell death) in cancer cells, inhibiting tumor growth, and modulating the tumor microenvironment. These properties make AMPs potential candidates for cancer therapy and drug development. AMPs exert selective control over the growth of commensal microorganisms, thereby shaping the composition of the host’s microbiota and contributing to the maintenance of a balanced microbial community. AMPs also act as important components of the innate immune systems of insects and other invertebrates by protecting against microbial invaders. The multifaceted biological functions of AMPs underscore their significance in host defense, immune regulation, and overall health across a variety of biological contexts.

**Figure 1:**
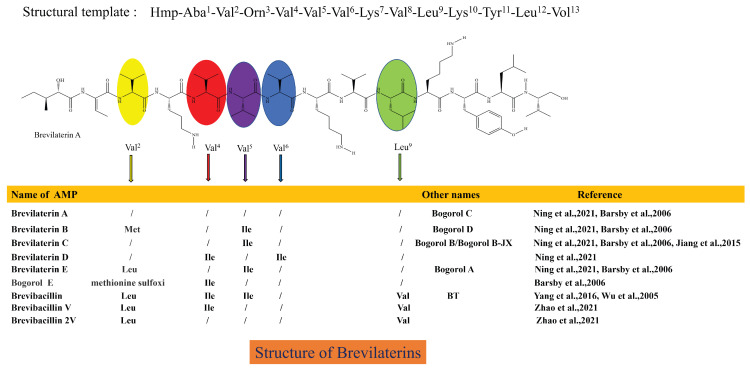
Structural characteristics of Brevilaterins [10,26,64,65,66,67]. This structure diagram has been drawn based on information regarding the structure and visual representation provided in reference [63,64]. In the same amino acid sequence template, the structures of these antimicrobial peptides differ only at the amino acids at specific positions, and the corresponding substituted amino acids are indicated by different colored ovals.

*B. laterosporus* produces a variety of AMPs and is thought to be responsible for producing short-sequence (<20 AAs) AMPs [10,62]. Seventeen AMPs produced by *B. laterosporus* have been identified, but the nomenclature varies among discoverers [63], including Bogorols [64], Brevibacillins [65,66], Brevilaterins [10], BT peptides [67], BL-A60 [25], Bacteriocin DS-3 [68], and others. Bogorols, Brevibacillins, Brevilaterins, and BT peptides have similar basic structures. They contain 13 amino acids and an N-terminal C6-fatty acid chain (Hmp-Aba-Val-Orn-Val-Val-Val-Lys-Val-Leu-Lys-Tyr-Leu-Vol) [63], with a molecular weight of 1.555–1.617 kDa, with amino acid substitutions at some positions. Therefore, these similar AMPs should be named Brevilaterins, as shown in Figure 1. *B. laterosporus* also synthesizes two other structurally different AMPs, which are BL-A60 and DS-3. Their amino acid sequences are CH-Leu-Tyr-Lys-Leu-Val-Lys-Val-Val-Leu-Asn-Met-TA (1.602 kDa) and Leu-Asn-Thr-Leu-Glu-Thr-Glu-Glu-Trp-Phe-Phe-Lys (1.593 kDa).

### 4.2. Mechanisms of Antimicrobial Actions of Brevilaterins

Brevilaterins (including Brevilaterin A-E and Brevibacillins) are antimicrobial lipopeptides isolated from *B. laterosporus* [10,65] that are natural antibacterial agents and cationic bacitracins against drug-resistant bacteria [11,66,69,70,71]. This lipopeptide maintained good stability at 121 °C and pH 2–12 [72]. The minimum inhibitory concentration of Brevilaterins against *Bacillus*, *Listeria*, *Streptococcus*, *Lactobacillus*, and other bacteria ranged from 0.5 to 2 μg/mL [69]. The broad spectrum and potent antibacterial effects of these AMPs render them promising candidates for antibiotic utilization.

**Figure 2:**
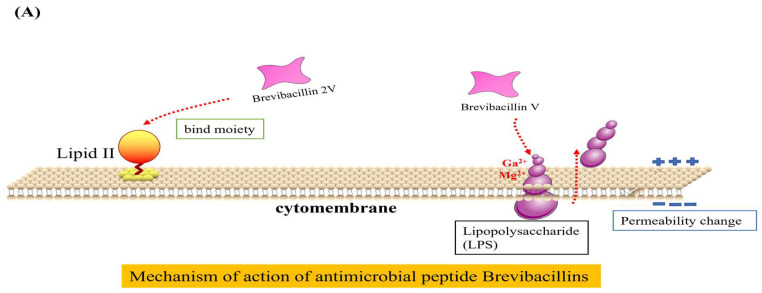

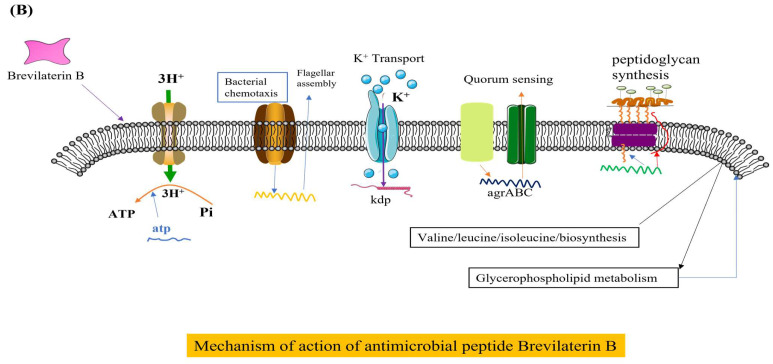
Diagram of the mechanism of action of Brevilaterins. The antibacterial mechanism diagram of Brevibacillins is illustrated in (**A**), based on the content information derived from references [11,73]. The antibacterial mechanism diagram of Brevilaterin B is illustrated in (**B**), which has been constructed based on the structural and pictorial information provided in reference [74].

The antibacterial mechanisms of Brevilaterins have been demonstrated at two distinct levels. At the cellular level, the cationic peptide Brevibacillin V interacted with negatively charged lipopolysaccharide (LPS) through electrostatic interactions. It replaced Mg^2+^ and Ca^2+^ ions that maintained the LPS structure, resulting in partial release of LPS into the environment [73]. The missing LPS was filled by phospholipids in the inner membrane, leading to increased permeability of the outer membrane. Subsequent investigations revealed that Brevibacillin 2V bound to the Lipid II pentapeptide, which is a precursor involved in bacterial cell wall synthesis, thereby changing the permeability of the bacterial cell membrane and exhibiting bactericidal activity [11], as illustrated in Figure 2A. The expression of the *murC*, *murY*, *murE*, and *murG* genes involved in cell wall synthesis were significantly downregulated by Brevilaterin B, resulting in a pronounced inhibition of peptidoglycan biosynthesis (*p* < 0.05). The expression levels of the *plcB*, *cls-1*, and *cls-2* genes associated with phosphatidylglycerol degradation and transformation were significantly upregulated (*p* < 0.05). ATP synthesis was impaired, resulting in a significant downregulation of transcriptional genes such as *atpB* (*p* < 0.05). Genes related to potassium ion transport and the potassium-regulating two-component system, including *kdpB*, were significantly upregulated (*p* < 0.05). Furthermore, expression of the stress response and of the cation resistance-related gene *dltA* were significantly upregulated. Brevilaterin B modulates gene expression in pathogen membrane-associated pathways. These pathways encompass activation of peptidoglycan biosynthesis, membrane transport (ATP-binding cassette transport and ion transport), cellular metabolism (amino acid and lipid metabolism), ATP synthesis, and the stress response (quorum sensing and bacterial chemotaxis) [74], as illustrated in Figure 2B, to achieve the antibacterial effect.

The AMPs produced by *B. laterosporus* exhibit extensive and potent antibacterial activity. They have been found to be highly effective against a wide range of bacteria, including both Gram-positive and Gram-negative species, making them a promising source of potential antibiotics. Additionally, these AMPs demonstrate excellent thermal stability, enabling them to withstand high temperatures without compromising their antimicrobial activity. Despite these promising characteristics, the precise mechanisms underlying the antibacterial effects of these AMPs remain incompletely understood. Further research and investigation are necessary to elucidate their modes of action in order to optimize their potential use as antibiotics and comprehend the broader implications of their activity in relation to bacterial resistance and the development of new antimicrobial agents. As our understanding of these AMPs continues to expand, we anticipate that future investigations will unveil previously unknown insights into the characteristics and mechanisms of other AMPs. Consequently, this will make a significant contribution to the ongoing endeavors in developing novel and effective antimicrobial agents—an imperative pursuit considering the escalating global challenge of antibiotic resistance. The more we acquire knowledge about the intricate mechanisms by which bacteria interact with their environment and other microorganisms, the better equipped we become in formulating targeted strategies for combating bacterial infections while preserving the efficacy of existing antibiotics.

## 5. Prospects for Industrializing *B. laterosporus*

*B. laterosporus* has diverse applications in agriculture, such as biological control, reducing reliance on chemical pesticides, and promoting crop growth [75]. *B. laterosporus* produces a diverse array of compounds with insecticidal activity, effectively controlling a wide range of pests including insects and nematodes. Utilizing biopesticides derived from *B. laterosporus* enables efficient management of agricultural pests while reducing reliance on chemical pesticides. Moreover, *B. laterosporus* is tolerant of abiotic stressors, aiding crops in adapting to environmental challenges. For example, the strain enhances tolerance to stress by regulating the synthesis and metabolism of endogenous hormones in response to challenging conditions, such as drought and salinity. Additionally, *B. laterosporus* plays a crucial role in the degradation of agricultural residues, as it possesses strong decomposition abilities that effectively breakdown chemical pesticide residues into non-toxic or minimally-toxic metabolites. This not only ensures food safety but alleviates pollution. The key application of *B. laterosporus* in agriculture lies in its utilization as a biocontrol agent for the management of pests and diseases during crop cultivation. However, due to its capacity for enhancing intestinal barrier function, regulating immunity, and inhibiting the proliferation of harmful bacteria, *B. laterosporus* has gradually been employed as a variety of animal feed additives in the breeding industry, demonstrating significant potential within the realm of animal production. Additionally, *B. laterosporus* facilitated the accelerated maturation of compost by regulating physicochemical parameters and orchestrating bacterial community succession, thereby providing valuable insights for the efficient utilization of manure resources and the integration of cultivation practices with sustainable development [76].

The practical application of *B. laterosporus* faces several challenges, including limited productivity, the absence of highly potent strains, and restricted industrial scalability. Firstly, one challenge lies in optimizing the production process for *B. laterosporus* on a larger scale. This entails developing efficient fermentation techniques capable of accommodating increased volumes while upholding high product quality and consistency. Secondly, strain optimization plays a crucial role in enhancing the efficacy and stability of it across various applications. This necessitates extensive research and development efforts focused on identifying genetic variations within different strains and selecting those exhibiting superior characteristics such as heightened antimicrobial activity or improved environmental adaptability. Further investigation is required to understand the genome and proteomics of *B. laterosporus*. Advancements in genomics and proteomics can significantly contribute to its practical application. Genomics can facilitate the identification of pivotal genes accountable for desirable traits in *B. laterosporus*, such as antimicrobial activity or stress tolerance, which can be exploited to genetically engineer strains with augmented characteristics. By analyzing the proteome of *B. laterosporus*, researchers can gain valuable insights into its metabolic pathways and protein functions, which are crucial for optimizing production processes and understanding how this bacterium interacts with its environment. Therefore, the following actions should be considered for successfully developing agricultural probiotic products based on *B. laterosporus*: (1) optimizing the formulation to enhance the stability and fermentation level of live bacterial preparations for improved production efficiency; (2) exploring highly active strains and enhancing the screening system; (3) utilizing mutation breeding or genetic engineering techniques to improve strains and increase effective metabolite yield; (4) continuously researching and developing new bioactive products with enhanced functionality for product enhancement.

## Figures and Tables

**Table 1:** Bacteriostatic or disease-control activity of *B. laterosporus* against pathogenic fungi in crops.

Strains	Pathogenic Fungi	Anti-Fungal Activity *	Reference
*B. laterosporus* ZQ2	*Rhizoctonia solani*,	Growth inhibition was 80.17%	[1]
*B. laterosporus* JX-5	*Botryosphaeria dothidea*	Biocontrol efficacy was 70%	[26]
*B. laterosporus* A60	*Phytophthora capsici*	Biocontrol efficacy was 96.55%	[25]
*B. laterosporus* Bl13	*Alternaria solani*	Biocontrol efficacy was 26.7%	[29]
*B. laterosporus* BPM3	*Magnaporthe grisea* Cav.	Biocontrol efficacy ranged from 30% to 67%	[30]
*B. laterosporus* K75	*Fusarium oxysporum*	Growth inhibition was 26%	[20]

* Growth inhibition (%) = [(mycelia length in the control plate—mycelia length in the treated plate)/mycelia length in the control plate × 100]. Disease index = ∑ (disease severity × number of corresponding disease severity tubers)/(highest disease severity × total number of tubers) × 100%, Biocontrol efficacy = (disease index of control—disease index of treated)/disease index of control × 100%.

**Table 2:** The regulation of animal growth mediated by *B. laterosporus*.

Strains	Species of Animals	Status of Improvement	Reference
*B. laterosporus* S62-9	arbor acre male broiler	Body weight exhibited a 7.2% increase, while the feed conversion rate (FCR) demonstrated a significant decrease of 5.19%, leading to an overall enhancement in production performance	[48]
*B. laterosporus* Texasporus	male broiler	The carcass percentage was increased by 1.11%, which improved the production performance	[49]
*B. laterosporus* PBC01	crucian carp	Weight gain rate (WGR) and specific growth rate (SGR) were significantly increased by 32.19% and 0.24%, and FCR was decreased by 0.28, which improved the production performance	[50]
*B. laterosporus* FAS05	*Litopenaeus vannamei*	SGR significantly increased by 0.5% (*p* < 0.05) to improve production performance	[51]
*B. laterosporus* S62-9	arbor acre male broiler	The meat quality of broilers could be enhanced through significant improvements in breast muscle color (0.5% decrease in lightness and 0.54% increase in redness) as well as muscle chemistry (0.05% increase in protein content)	[52]

## Data Availability

No new data were created or analyzed in this study. Data sharing is not applicable to this article.

## References

[1] Song Z., Liu K., Lu C., Yu J., Ju R., Liu X. (2011). Isolation and characterization of a potential biocontrol *Brevibacillus laterosporus*. Afr. J. Microbiol. Res..

[2] Hassi M., El Guendouzi S., Haggoud A., David S., Ibnsouda S., Houari A., Iraqui M. (2012). Antimycobacterial activity of a *Brevibacillus laterosporus* strain isolated from a Moroccan soil. Braz. J. Microbiol..

[3] Suslova M.Y., Lipko I., Mamaeva E., Parfenova V. (2012). Diversity of cultivable bacteria isolated from the water column and bottom sediments of the Kara Sea shelf. Microbiology.

[4] Khaled J.M., Al-Mekhlafi F.A., Mothana R.A., Alharbi N.S., Alzaharni K.E., Sharafaddin A.H., Kadaikunnan S., Alobaidi A.S., Bayaqoob N.I., Govindarajan M. (2018). *Brevibacillus laterosporus* isolated from the digestive tract of honeybees has high antimicrobial activity and promotes growth and productivity of honeybee’s colonies. Environ. Sci. Pollut. Res..

[5] Wang Z.-X., Zhang D., Li X.-F., Ning Y.-W., Zhang F.-J., Jia Y.-M. (2016). A Study on the Biological Characteristics of *Brevibacillus laterosporus* S62-9. Mod. Food Sci. Technol..

[6] Smirnova T., Minenkova I., Orlova M., Lecadet M., Azizbekyan R. (1996). The crystal-forming strains of *Bacillus laterosporus*. Res. Microbiol..

[7] Laubach C. (1916). Studies on aerobic spore-bearing non-pathogenic bacteria Part II spore-bearing bacteria in dust. J. Bacteriol..

[8] Shida O., Takagi H., Kadowaki K., Komagata K. (1996). Proposal for two new genera, *Brevibacillus* gen. nov. and *Aneurinibacillus* gen. nov. Int. J. Syst. Evol. Microbiol..

[9] Ruiu L. (2013). *Brevibacillus laterosporus*, a pathogen of invertebrates and a broad-spectrum antimicrobial species. Insects.

[10] Ning Y., Han P., Ma J., Liu Y., Fu Y., Wang Z., Jia Y. (2021). Characterization of brevilaterins, multiple antimicrobial peptides simultaneously produced by *Brevibacillus laterosporus* S62-9, and their application in real food system. Food Biosci..

[11] Zhao X., Wang X., Shukla R., Kumar R., Weingarth M., Breukink E., Kuipers O.P. (2021). Brevibacillin 2V Exerts Its Bactericidal Activity via Binding to Lipid II and Permeabilizing Cellular Membranes. Front. Microbiol..

[12] Prasanna L., Eijsink V.G., Meadow R., Gseidnes S. (2013). A novel strain of *Brevibacillus laterosporus* produces chitinases that contribute to its biocontrol potential. Appl. Microbiol. Biotechnol..

[13] Anbu P. (2016). Enhanced production and organic solvent stability of a protease from *Brevibacillus laterosporus* strain PAP04. Braz. J. Med. Biol. Res..

[14] Chen Z., Wang L., Liu Y., Han P., Hong D., Li S., Ma A., Jia Y. (2022). Brevilaterin B from *Brevibacillus laterosporus* has selective antitumor activity and induces apoptosis in epidermal cancer. World J. Microbiol. Biotechnol..

[15] Pii Y., Mimmo T., Tomasi N., Terzano R., Cesco S., Crecchio C. (2015). Microbial interactions in the rhizosphere: Beneficial influences of plant growth-promoting rhizobacteria on nutrient acquisition process. A review. Biol. Fertil. Soils.

[16] Masciarelli O., Llanes A., Luna V. (2014). A new PGPR co-inoculated with Bradyrhizobium japonicum enhances soybean nodulation. Microbiol. Res..

[17] Suping M. (2016). Effects of Different Concentrations of Bacillus Subtilis on Soil and Crop Growth. J. Jining Univ..

[18] Zhu J., Zhang S., Guo J., Gao C., Tian W., Zhou B. (2014). Application Effects of *Brevibacillus laterosporus* (AMCC 100018) on Greenhouse Cucumber. J. Chang. Veg..

[19] Fitriatin B.N., Arief D.H., Simarmata T., Santosa D.A., Joy B. (2011). Phosphatase-producing bacteria isolated from Sanggabuana forest and their capability to hydrolyze organic phosphate. J. Soil Sci. Environ. Manag..

[20] Świątczak J., Kalwasińska A., Wojciechowska A., Brzezinska M.S. (2023). Physiological properties and genomic insights into the plant growth—Promoting rhizobacterium *Brevibacillus laterosporus* K75 isolated from maize rhizosphere. J. Sci. Food Agric..

[21] Nehra V., Saharan B.S., Choudhary M. (2016). Evaluation of Brevibacillus brevis as a potential plant growth promoting rhizobacteria for cotton (*Gossypium hirsutum*) crop. Springerplus.

[22] Wang X., Zhang J., Wang X., An J., You C., Zhou B., Hao Y. (2022). The growth-promoting mechanism of *Brevibacillus laterosporus* AMCC100017 on apple rootstock Malus robusta. Hortic. Plant J..

[23] Chen S., Zhang M., Wang J., Lv D., Ma Y., Zhou B., Wang B. (2017). Biocontrol effects of *Brevibacillus laterosporus* AMCC100017 on potato common scab and its impact on rhizosphere bacterial communities. Biol. Control.

[24] Nega A. (2014). Review on concepts in biological control of plant pathogens. J. Biol. Agric. Healthc..

[25] Zhao J., Guo L., Zeng H., Yang X., Yuan J., Shi H., Xiong Y., Chen M., Han L., Qiu D. (2012). Purification and characterization of a novel antimicrobial peptide from *Brevibacillus laterosporus* strain A60. Peptides.

[26] Jiang H., Wang X., Xiao C., Wang W., Zhao X., Sui J., Sa R., Guo T.L., Liu X. (2015). Antifungal activity of *Brevibacillus laterosporus* JX-5 and characterization of its antifungal components. World J. Microbiol. Biotechnol..

[27] Yang Z., Xu D., Du C. (2014). Studies on fermentation conditions of antimicrobial substances produced by *Brevibacillus laterosporus* BL-21. Agric. Sci. Technol..

[28] Zayed M., El-Garawani I.M., El-Sabbagh S.M., Amr B., Alsharif S.M., Tayel A.A., AlAjmi M.F., Ibrahim H.M., Shou Q., Khalifa S.A. (2022). Structural Diversity, LC-MS-MS Analysis and Potential Biological Activities of *Brevibacillus laterosporus* Extract. Metabolites.

[29] Sun Y., Liu Z., Li H., Zheng Z., Ji C., Guo Q., Lai H. (2021). Biocontrol effect and mechanism of *Bacillus laterosporus* Bl13 against early blight disease of tomato. Ying Yong Sheng Tai Xue Bao = J. Appl. Ecol..

[30] Saikia R., Gogoi D., Mazumder S., Yadav A., Sarma R., Bora T., Gogoi B. (2011). *Brevibacillus laterosporus* strain BPM3, a potential biocontrol agent isolated from a natural hot water spring of Assam, India. Microbiol. Res..

[31] Raaijmakers J.M., Mazzola M. (2012). Diversity and natural functions of antibiotics produced by beneficial and plant pathogenic bacteria. Annu. Rev. Phytopathol..

[32] Li W., Jiang R. (2007). Identification and field application of antagonistic strain X10 against tomato bacterial wilt. Soil Fertil. Sci. China.

[33] Li C., Shi W., Wu D., Tian R., Wang B., Lin R., Zhou B., Gao Z. (2021). Biocontrol of potato common scab by Brevibacillus laterosporus BL12 is related to the reduction of pathogen and changes in soil bacterial community. Biol. Control.

[34] Su X.-x., Wan T.-t., Gao Y.-d., Zhang S.-h., Chen X., Huang L.-q., Wang W. (2024). Action mechanism of the potential biocontrol agent *Brevibacillus laterosporus* SN19-1 against Xanthomonas oryzae pv. oryzae causing rice bacterial leaf blight. Arch. Microbiol..

[35] Kakar K.U., Nawaz Z., Cui Z., Almoneafy A.A., Zhu B., Xie G.-L. (2014). Characterizing the mode of action of *Brevibacillus laterosporus* B4 for control of bacterial brown strip of rice caused by A. avenae subsp. avenae RS-1. World J. Microbiol. Biotechnol..

[36] Glare T.R., Hampton J.G., Cox M.P., Bienkowski D.A. (2016). Novel strains of Brevibacillus laterosporus as biocontrol agents against plant pests, particularly Lepidoptera and Diptera.

[37] Khanna K., Kohli S.K., Ohri P., Bhardwaj R. (2021). Plants-nematodes-microbes crosstalk within soil: A trade-off among friends or foes. Microbiol. Res..

[38] Zheng Z., Zheng J., Zhang Z., Peng D., Sun M. (2016). Nematicidal spore-forming Bacilli share similar virulence factors and mechanisms. Sci. Rep..

[39] Hamze R., Ruiu L. (2022). *Brevibacillus laterosporus* as a natural biological control agent of soil-dwelling nematodes. Agronomy.

[40] Huang X., Tian B., Niu Q., Yang J., Zhang L., Zhang K. (2005). An extracellular protease from *Brevibacillus laterosporus* G4 without parasporal crystals can serve as a pathogenic factor in infection of nematodes. Res. Microbiol..

[41] Tian B., Li N., Lian L., Liu J., Yang J., Zhang K.-Q. (2006). Cloning, expression and deletion of the cuticle-degrading protease BLG4 from nematophagous bacterium *Brevibacillus laterosporus* G4. Arch. Microbiol..

[42] Barbieri G., Ferrari C., Mamberti S., Gabrieli P., Castelli M., Sassera D., Ursino E., Scoffone V.C., Radaelli G., Clementi E. (2021). Identification of a Novel *Brevibacillus laterosporus* Strain with Insecticidal Activity against Aedes albopictus Larvae. Front. Microbiol..

[43] Bedini S., Muniz E.R., Tani C., Conti B., Ruiu L. (2020). Insecticidal potential of *Brevibacillus laterosporus* against dipteran pest species in a wide ecological range. J. Invertebr. Pathol..

[44] Carramaschi I.N., Pereira L.d.A., Queiroz M.M.d.C., Zahner V. (2015). Preliminary screening of the larvicidal effect of *Brevibacillus laterosporus* strains against the blowfly Chrysomya megacephala (Fabricius, 1794)(Diptera: Calliphoridae). Rev. Soc. Bras. Med. Trop..

[45] Marche M.G., Mura M.E., Falchi G., Ruiu L. (2017). Spore surface proteins of *Brevibacillus laterosporus* are involved in insect pathogenesis. Sci. Rep..

[46] Schnepf H.E., Narva K.E., Stockhoff B.A., Lee S.F., Walz M., Sturgis B. (2010). Pesticidal Toxins and Genes from Bacillus Laterosporus Strains.

[47] Kouadio J.-L., Duff S., Aikins M., Zheng M., Rydel T., Chen D., Bretsnyder E., Xia C., Zhang J., Milligan J. (2021). Structural and functional characterization of Mpp75Aa1.1, a putative beta-pore forming protein from *Brevibacillus laterosporus* active against the western corn rootworm. PLoS ONE.

[48] Zhi T., Ma A., Liu X., Chen Z., Li S., Jia Y. (2023). Dietary Supplementation of *Brevibacillus laterosporus* S62-9 Improves Broiler Growth and Immunity by Regulating Cecal Microbiota and Metabolites. Probiotics Antimicrob. Proteins.

[49] Purba M., Sepriadi S., Trisna A., Desnamrina K., Hua L. (2022). The effect of *Brevibacillus laterosporus* texasporus culture on percentage of carcass broilers chickens infected with salmonella pullorum. Proceedings of the IOP Conference Series: Earth and Environmental Science.

[50] Yang D., Wang Z., Dai X., Liu M., Zhang D., Zeng Y., Zeng D., Ni X., Pan K. (2023). Addition of *Brevibacillus laterosporus* to the rearing water enhances the water quality, growth performance, antioxidant capacity, and digestive enzyme activity of crucian carp Carassius auratus. Fish. Sci..

[51] Daode Y., Kaikai L., Jingjing S., Shaojing G., Ancheng Z., Xiaolu W., Ying F., Youhong W., Hongjun L. (2023). Effects of Adding Brevibacillus laterosporu FAS05 to Feed on the Growth, Disease Resistance, and Immunity of Litopenaeus vannamei. Prog. Fish. Sci..

[52] Liu X., Ma A., Zhi T., Hong D., Chen Z., Li S., Jia Y. (2023). Dietary Effect of *Brevibacillus laterosporus* S62-9 on Chicken Meat Quality, Amino Acid Profile, and Volatile Compounds. Foods.

[53] Jiang Y. (2022). BT Lipopeptides Are Used as Therapeutic Agents for Obesity and Related Diseases.

[54] Chen C., Li J., Zhang H., Xie Y., Xiong L., Liu H., Wang F. (2020). Effects of a probiotic on the growth performance, intestinal flora, and immune function of chicks infected with Salmonella pullorum. Poult. Sci..

[55] Weng G., Huang J., Ma X., Song M., Yin Y., Deng D., Deng J. (2022). *Brevibacillus laterosporus* BL1, a promising probiotic, prevents obesity and modulates gut microbiota in mice fed a high-fat diet. Front. Nutr..

[56] Bagherzadeh Kasmani F., Torshizi K., Mehri M. (2018). Effect of *Brevibacillus laterosporus* probiotic on hematology, internal organs, meat peroxidation and ileal microflora in Japanese quails fed aflatoxin B1. J. Agric. Sci. Technol..

[57] Bahar A.A., Ren D. (2013). Antimicrobial peptides. Pharmaceuticals.

[58] Erdem Büyükkiraz M., Kesmen Z. (2022). Antimicrobial peptides (AMPs): A promising class of antimicrobial compounds. J. Appl. Microbiol..

[59] Olga K., Marina K., Alexey A., Anton S., Vladimir Z., Igor T. (2020). The role of plant antimicrobial peptides (AMPs) in response to biotic and abiotic environmental factors. Biol. Commun..

[60] Starr C.G., Maderdrut J.L., He J., Coy D.H., Wimley W.C. (2018). Pituitary adenylate cyclase-activating polypeptide is a potent broad-spectrum antimicrobial peptide: Structure-activity relationships. Peptides.

[61] Luo Y., Song Y. (2021). Mechanism of antimicrobial peptides: Antimicrobial, anti-inflammatory and antibiofilm activities. Int. J. Mol. Sci..

[62] Jiang H., Ji C., Sui J., Sa R., Wang X., Liu X., Guo T.L. (2017). Antibacterial and antitumor activity of Bogorol B-JX isolated from *Brevibacillus laterosporus* JX-5. World J. Microbiol. Biotechnol..

[63] Han P., Ma A., Ning Y., Chen Z., Liu Y., Liu Z., Li S., Jia Y. (2023). Global gene-mining strategy for searching nonribosomal peptides as antimicrobial agents from microbial sources. LWT.

[64] Barsby T., Warabi K., Sørensen D., Zimmerman W.T., Kelly M.T., Andersen R.J. (2006). The bogorol family of antibiotics: Template-based structure elucidation and a new approach to positioning enantiomeric pairs of amino acids. J. Org. Chem..

[65] Yang X., Huang E., Yuan C., Zhang L., Yousef A.E. (2016). Isolation and structural elucidation of brevibacillin, an antimicrobial lipopeptide from *Brevibacillus laterosporus* that combats drug-resistant Gram-positive bacteria. Appl. Environ. Microbiol..

[66] Zhao X., Wang X., Shukla R., Kumar R., Weingarth M., Breukink E., Kuipers O.P. (2021). Brevibacillin 2V, a novel antimicrobial lipopeptide with an exceptionally low hemolytic activity. Front. Microbiol..

[67] Wu X., Ballard J., Jiang Y.W. (2005). Structure and biosynthesis of the BT peptide antibiotic from Brevibacillus texasporus. Appl. Environ. Microbiol..

[68] Odah K.A., Dong W.-L., Lei L., Atiah L.A., Wang Y.-m., Kong L.-C., Ma H.-X. (2020). Isolation, identification, and characterization of a novel bacteriocin produced by *Brevibacillus laterosporus* DS-3 against methicillin-resistant Staphylococcus aureus (MRSA). Int. J. Pept. Res. Ther..

[69] Liu Y., Han P., Jia Y., Chen Z., Li S., Ma A. (2022). Antibacterial regularity mining beneath the systematic activity database of lipopeptides Brevilaterins: An instructive activity handbook for its food application. Foods.

[70] Yang X., Huang E., Yousef A.E. (2017). Brevibacillin, a cationic lipopeptide that binds to lipoteichoic acid and subsequently disrupts cytoplasmic membrane of Staphylococcus aureus. Microbiol. Res..

[71] Wu Y., Zhou L., Lu F., Bie X., Zhao H., Zhang C., Lu Z., Lu Y. (2019). Discovery of a novel antimicrobial lipopeptide, brevibacillin V, from *Brevibacillus laterosporus* fmb70 and its application on the preservation of skim milk. J. Agric. Food Chem..

[72] Chen Z., Wang X., Han P., Liu Y., Hong D., Li S., Ma A., Jia Y. (2022). Discovery of novel antimicrobial peptides, Brevilaterin V, from *Brevibacillus laterosporus* S62-9 after regulated by exogenously-added L-valine. LWT.

[73] Wu Y., Nie T., Meng F., Zhou L., Chen M., Sun J., Lu Z., Lu Y. (2021). The determination of antibacterial mode for cationic lipopeptides brevibacillins against Salmonella typhimurium by quantum chemistry calculation. Appl. Microbiol. Biotechnol..

[74] Liu Y., Ning Y., Chen Z., Han P., Zhi T., Li S., Ma A., Jia Y. (2023). Transcriptomics reveals substance biosynthesis and transport on membranes of Listeria monocytogenes affected by antimicrobial lipopeptide brevilaterin B. Food Sci. Hum. Wellness.

[75] Fang L., Hongqu W., Shaohua W., Wei F., Kaimei W. (2023). The Application Potential of *Brevibacillus laterosporus* in Agriculture. Chin. J. Biol. Control.

[76] Ren L., Li J., Li H., Guo Z., Li J., Lv Y. (2023). Inoculating exogenous bacterium *Brevibacillus laterosporus* ZR-11 at maturity stage accelerates composting maturation by regulating physicochemical parameters and indigenous bacterial community succession. Environ. Sci. Pollut. Res..

